# Neiguan and Jianshi Acupoint Stimulation Aids Hemodynamic Stability in a Cervical Cord Trauma Patient

**DOI:** 10.5812/atr.8009

**Published:** 2012-10-14

**Authors:** Shalini T Adhikari, Mohammed Juma Suliman Al-Nabi, Neelam Suri, Rashid M Khan, Naresh K Kaul

**Affiliations:** 1 Department of Anesthesia and ICU, National Trauma Centre, Khoula Hospital, Muscat, Oman

**Keywords:** Acupuncture, Hypotension, Shock, Hemodynamic

## Abstract

A 36-year-old male patient with posttraumatic cervical cord damage and resultant quadriparesis, demonstrated hypotension and periods of bradycardia. For most of his two-month stay in the Intensive Care Unit (ICU), he was dependent on dopamine support to maintain hemodynamic stability. Keeping in mind evidence from the literature, that electrostimulation of acupoints Neiguan (PC - 6) and Jianshi (PC - 5) has therapeutic efficacy in restoring hypotension, we treated this patient with two six-hour periods of electrostimulation at these acupoints. We noted beneficial hemodynamic effects, with a resultant successful withdrawal of dopamine support lasting for up to 48 hours. This case report demonstrates the therapeutic efficacy of electrostimulation of PC - 5 and PC - 6 acupoints to wean a patient off chronic dopamine support, and this warrants further investigation.

## 1. Background

Hypotension and hemodynamic instability is a frequently encountered clinical problem in the intensive care setting. When mean arterial blood pressure falls below approximately 60 mm Hg, end-organ perfusion becomes compromised. The cornerstones of management include volume resuscitation and therapy directed toward the underlying causes of the hypotension. When these measures fail to restore blood pressure and vital organ perfusion, the administration of intravenous vasoactive agents may become necessary (1). It has been well accepted that Chinese based acupuncture techniques at the Neiguan (PC - 6) and Jianshi (PC - 5) points influence vascular pressor responses and the cardiovascular sympathetic system (2, 3). In particular, electrostimulation of the PC-6 point has been shown to increase cardiovascular variables such as stroke volume and cardiac output and it also alleviates hemorrhage induced hypotension (4, 5). More recently, Arai et al. demonstrated that transcutaneous electrical nerve stimulation (TENS) at the PC - 5 and PC - 6 acupoints reduced the severity of hypotension after spinal anesthesia in patients who underwent a caesarean section. They observed that more ephedrine was required to maintain arterial blood pressure in the control and non-acupoint groups, compared with the acupoint stimulation group (6). We recently had a 36-year-old male quadri paretic patient in our Intensive Care Unit (ICU), who had been involved in a road traffic accident. Over the last two months of his stay in the ICU, he had been on dopamine support intermittently. This resulted in the patient becoming dopamine dependent and he would become hypotensive with any attempt to wean him off the drug. Contrary to any serious expectations, we subjected the patient to electro stimulation at the PC - 5 and PC - 6 points with gratifying results in restoring a short period of hemodynamic stability without dopamine support.

## 2. Case Report

**Figure 1.**
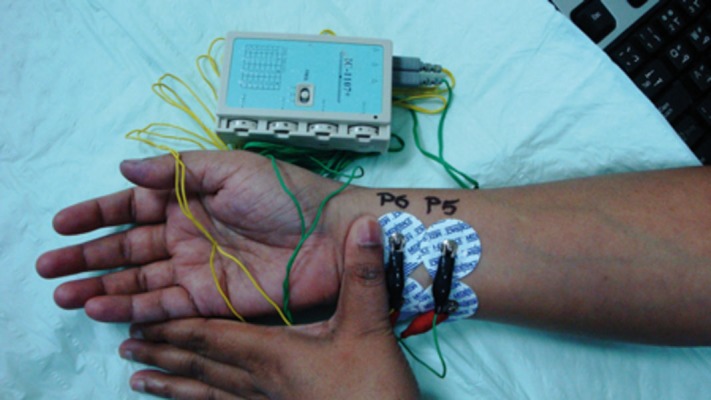
Application of Electrodes to PC - 6 and PC - 5 Acupoints 2 Cun (Equivalent to the Interphalangeal Width of the Patient’s Thumb) Proximal to the Anterior Crease of the Wrist on the Palmar Side of the Arm, Between the Tendons of the Palmaris Longus Muscle and Flexor Carpi Radialis

A 36-year-old male patient with quadriparesis, following a road traffic accident, had undergone an anterior corpectomy C5 with fusion of C4 - 6. For a period of over two months he was off and on a ventilator. Over the last ten day period, he had become dependent on low dose dopamine (4 - 6 µg/kg/min) to maintain hemodynamic stability. During his initial stay in the ICU, he had also showed periods of bradycardia for which a temporary pace maker was inserted. Over the last month this was withdrawn. A percutaneous tracheostomy was performed so that he could be shifted to the ward. The patient subsequently underwent a successful trial of spontaneous breathing on a ‘T’ piece with a fraction of inspired oxygen (FiO2) of 0.35. Though the patient was otherwise doing well (blood pressure, heart rate, and central venous pressure ranging between 107/61 to 91/58, 47/min to 81/min, and 3 to 7 cm H_2_O respectively), he was still dependent on low dose dopamine to stabilize his vitals. Unfortunately, any attempt at weaning him from dopamine would result in hypotension, despite optimal hydration, and two three-day periods of hydrocortisone (50mg 6 hourly). It was then suggested to give him a trial of six hours of transcutaneous electrical nerve stimulation (TENS) at PC - 5 and PC - 6 point (6). At this point, the patient’s blood pressure was 106/66, with a heart rate of 88/min. After noting initial vital parameters, a PC - 6 point electrode was placed 2 cun (equivalent to the interphalangeal width of the patient’s thumb) proximal to the anterior crease of the wrist, on the palmar side of the arm, between the tendons of the palmaris longus muscle, and flexor carpi radialis. A second electrode was placed 2 cm lateral to the PC-6 point electrode to allow an electrical current to flow through the acupoint. A PC - 5 electrode was placed 2 cm proximal to the PC-6 electrode and likewise another electrode 2 cm lateral to it (Figure 1). These two acupoints were stimulated in this patient using a TENS stimulator (IC- 1107+, Ito Co., Ltd, Japan). The intensity of the electrical stimulation was adjusted to the level which just failed to produce muscle contractions at a frequency of 40Hz. The electrical stimulation was delivered for six hours. After the first two hours the patient’s blood pressure started to rise marginally, which prompted us to start reducing inotropic support from; 6 µg/kg/min to 4 µg/kg/min. This rate was maintained for the next two hours and was there after withdrawn altogether. Two hours later, acupoint electrostimulation was also withdrawn. It was noted that the patient was maintaining his blood pressure between 102/60 to 112/76 mmHg with a heart rate ranging between 82-90/min. However, other objective monitoring of the effects of the electrostimulation such as an echocardiography was not done. This period of hemodynamic stability lasted for seven hours, thereafter it once again started declining gradually. Before his blood pressure fell below 90/66, electrostimulation was started once again and continued for the next six hours. The patient’s blood pressure again rose and settled at the previous stable values. His vitals remained stable for the next 24 hours and the patient could then be transferred to the ward without dopamine support.

Unfortunately, the patient’s respiration and hemodynamics again deteriorated in the ward and he had to be transferred back to the ICU after three days. The patient remained in and out of the ICU for the next three months without any further electrostimulation trials. Ultimately, he developed sepsis and expired six months after sustaining the road traffic accident.

## 3. Discussion

Patients with cervical cord injuries carry a poor prognosis. Hemodynamic instability is not uncommon in these patients. These patients are known to develop neurogenic shock secondary to sympathetic inactivation as their cardiac functions are controlled by the cranial nerves. This results in systemic hypotension and bradycardia (7). Several other factors have been recognized which may also play a role in arterial hypotension and bradycardia. These include low circulating plasma catecholamines, impaired baroreceptor function, morphologic changes in sympathetic neurons and loss of skeletal muscle pumping activity (8). These patients often need vasoactive support to stabilize their cardiovascular systems. Dopamine is particularly useful in patients who exhibit both hypotension, which is unresponsive to fluid resuscitation, and periods of bradycardia (7), like the present patient. Syuu et al. observed in their study on hypotensive mongrel dogs, secondary to controlled hemorrhage, that PC-6 electrostimulation was helpful in improving left ventricular filling. They attributed this to increased venous return as a result of enhanced vasomotor tone and muscle pumping. Interestingly, they noted that non-acupoint electrostimulation was relatively ineffective (5). Our quadriparetic patient with partial loss of skeletal muscle pump activity could have been helped by this property of PC-6 electrostimulation. In yet another study by Syuu et al., it was observed that PC-6 electrostimulation resulted in an increase in mean arterial pressure, heart rate, stroke volume and cardiac output by 10 - 15% when applied to open chest dog models (4). This effect could also have been responsible for the dopamine sparing effect by PC-6 electrostimulation in our patient. Unfortunately, none of the earlier studies showed sustained efficacy of acupoint electrostimulation in restoring hemodynamic stability (4, 5). Though the response of the present patient to acupoint electrostimulation demonstrated the effectiveness of this modality of managing a vasopressor-dependent, hypotensive cervical cord trauma patient, the response was again transient. The observation of positive hemodynamic effects following electrostimulation of acupoints in our patient suggests several advantages of this modality of patient management. First, although a TENS machine may not be readily available in all institutions, a peripheral nerve stimulator (PNS) can easily be procured from operating theatres to provide acupoint electrostimulation. A PNS has been successfully used as an alternative to a TENS machine for stimulating PC - 6 acupoint (9). Thus, the use of this type of electrostimulation modality would not incur any new expenditure on the institution. Second, the technique harnesses the body’s endogenous resources to stabilize hemodynamic levels and thus avoids the harmful effects of exogenous vasopressors. Lastly, it may be an acceptable addition to our current concept of multimodal approaches to patient management. In conclusion, this case report demonstrates the therapeutic efficacy of electrostimulation of PC-5 and PC-6 acupoints in patients with traumatic quadriparesis to wean them off chronic dopamine support and suggests the need for further investigations with larger sample sizes.
